# Parameter-Free and Electron Counting Satisfied Material Representation for Machine Learning Potential Energy and Force Fields

**DOI:** 10.1021/acs.jpclett.3c03250

**Published:** 2024-02-02

**Authors:** Bin Xi, Man Kit Chan, Kejie Bao, Wenjing Zhao, Ho Ming Chan, Hang Chen, Junyi Zhu

**Affiliations:** Department of Physics, The Chinese University of Hong Kong, Shatin, New Territory, Hong Kong SAR 999077, P.R. China

## Abstract

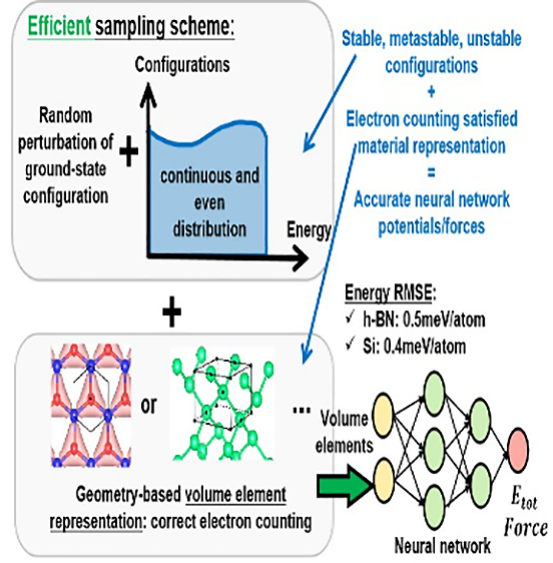

We proposed a parameter-free volume element representation that satisfies the electron counting model and obtains accurate machine learning potential energy and direct force fitting of randomly perturbed hexagonal BN. Our method preserves permutational, translational, and rotational invariance and can be extended to three-dimensional systems, verified by a system of bulk Si. As a result, we obtained 0.57 meV/atom potential energy root mean squared error (RMSE) and 59 meV/Å force RMSE for perturbed bulk BN systems and 0.43 meV/atom potential energy RMSE and 36 meV/Å force RMSE for perturbed Si systems. In addition, an unbiased perturbation-based data set construction scheme is introduced and a continuous population distribution is obtained with a training data set of 4500, which is about 1 order of magnitude smaller than standard methods based on first-principles molecular dynamics simulations and saves a large amount of computing resources. General validity of our model is verified by structure optimization, molecular dynamics simulations, and extrapolations.

First-principles methods-based materials simulations^1−6^ yield accurate results yet require heavy computational resources. Potential energy surface approaches based on empirical interatomic potentials^7−19^ are fast, however, often inaccurate. Recently, machine learning (ML) techniques^20−32^ with density functional theory (DFT)^33^ results as inputs yield fast simulations and accurate results. One popular choice is the descriptor-based neural network (NN) approaches^23,24,27−29,34^ by regarding the energy as a function of bond lengths, bond angles, or related symmetry functions.^23,27−29,35,36^ However, there are three main issues: (1) large amount of symmetry functions, predetermined subjectively; (2) time-consuming and poor accuracy in force fitting, based on gradient of energy; (3) no objective distribution criteria in the training data generation.

Specifically, the first proposal is based on a high-dimensional descriptor-based NN^23^ that represents atomic coordinates by a set of atom-centered symmetry functions, which encode atomic environments of each atom by two- and three-body terms with a lot of extra parameters.^23,35^ Recent development can be considered as its variations.^27−29,34,36−40^ However, the symmetry functions are usually predesigned intuitively and subjective for different material systems and the data processing time is long because of the extra parameters.^23,34,35^ Another issue is that such local bonding geometry does not contain electron counting information; however, the conservation of total electrons is the most common and important constraint for the many-body Schrodinger equation.^41^

Thus, a local sampling unit in the input data, incorporated with correct counting of local electrons, can be objective, interesting, and physical. The smallest possible sampling units for perfect crystals are primitive cells for bulk compounds. In addition, for crystals with perturbations, we can use the geometry of perturbed primitive cells as the input units, because the charge density of such units should be quite local. According to the Hohenberg–Kohn theorem,^42^ for a particular arrangement of external potential of the cell, the energy functional of the electrons in the cell can be minimized by the ground-state charge density distribution of the cell. Therefore, the localized ground-state density of the cell is expected to be the leading term of the ground-state energy of the cell, with high order terms of long-range interaction energy among electrons in different cells largely reduced and (or) easily fitted by the NN. Additionally, force predictions based on the gradient method suffer from numerical error and long computing time. Therefore, a local sampling unit approach for forces is also attractive, although a unit that is sufficient for accurate energy fitting may not be sufficient for the force fitting.

Another important issue is about the construction of a high-quality reference data set because ML models may become less capable of extrapolations of unseen data. Thus, training an NN requires a proper sampling in the configurational space that should cover as many perturbed states as possible that may occur in the first-principles MD simulations. A configurational sampling relying on MD trajectories or energetic local minima from DFT calculations is highly sparse and may be insufficient to reflect the actual molecular motion. Recently, sample-oriented methods are frequently used to govern the data quantity.^43^ For example, the idea of active learning^44−46^ is adopted, which performs an iterative process to gradually enlarge the data set size until convergence is achieved.^21,45^ However, the data set may be highly sensitive to the initial sampling pool and remain biased and incomplete. Also, many of these approaches are based on samples of structure optimization obtained from the DFT calculations, which are local minima of the PES and a large amount of nonlocal minimum configurations should be included but missed. To include these intermediate configurations, a systematic random perturbation of ions near the minima configuration of the PES can be effective and unbiased. One possible approach is to perturb a ground-state configuration randomly to capture possible configurations with a continuous population distribution as a function of the total energy (see later discussion for detailed data construction approach). More importantly, according to the Hohenberg–Kohn theorem, once the arrangement of the ionic positions is determined, the corresponding electronic ground state can be determined by the external potential of these ions, regardless of whether ions are arranged at local minimum structures.

In this paper, we proposed a new sampling unit of volume element (VE) representation of material systems (see later in the paper for detailed descriptions of VE). Such sampling units satisfy the conservation of total electrons without extra parameters such as radius cutoffs and can yield accurate potential energy and direct force predictions from a well-trained ML model. In addition, we proposed a reference data set construction approach based on random perturbations of ground-state configuration. Such a method enables us to capture as many configurations as possible without bias and results in a balanced and continuous configurational distribution in energy space, which is of crucial importance for training a plausible ML model. We demonstrate the success of both the representation and data construction approaches by accurate potential energy and force predictions of perturbed neutral hexagonal boron nitride (h-BN) bulk materials. Such a representation is also valid for three-dimensional materials, and we show an example of bulk silicon. In addition, VE representation preserves permutation, translational, and rotational invariance and ensures ML model scalability and flexibility for systems with a high degree of freedom with arbitrary shape at a low computational cost, which increases linearly with the number of atoms. Our code is available at https://github.com/binxi0629/VolumeElementPotentials.

Two principles should be satisfied during the construction of local sampling units as general descriptors for a perfect or perturbed bulk material. (1) All local sampling units together should fill up the entire space, and the electron counting model (ECM)^47^ should be satisfied in each sampling unit so that the ECM can be satisfied in the whole system. This principle is defined as a self-consistency principle. Note that, for systems with electronic dopants or surfaces that do not satisfy ECM locally, it may require extra treatments of charged states and a redefinition of large sampling units and is out of the scope of this paper. Also, for systems that can be regarded as perturbations of secondary phases, this principle should also apply with a different definition of the sampling unit. (2) A proximate-symmetric principle should be applied so that the influence of ions on or out of the boundary of the unit to the inner atom(s) should be approximately equal in a randomly perturbed system. Following these two principles, the unit should contain a center atom and other atoms surrounding the center one, so that a mapping between an input of a geometric representation of the unit and an output of energy of the unit or force of the center atom can be constructed in the ML model. We define such a representation as a VE. Note that the VE should include both the coordinates of all atoms in the unit and the additional coordinates that define the boundary of the unit. To satisfy the ECM, the smallest sampling unit should contain a minimum number of atoms that are equivalent to one chemical formula. At the same time, the coordinates of the VE give a distribution of the external potential that yields the leading term of the electronic ground state of the VE.

Next, we take bulk materials of h-BN and bulk Si as two examples to illustrate the details. In principle, this method should be valid for any two-dimensional system and also be extended to other three-dimensional systems with perturbations. For h-BN, a typical VE is illustrated by the black hexagon *aebfdg* with *o* at the center in Figure 1a. Note that the three vertices (*a*, *b*, and *d*) show the coordinates of three B atoms, that are the first-nearest-neighbors of the N atom at the center site of *o*, while the other three (*e*, *f*, and *g*) are the geometric centers of three nearby hexagons (*aobhij*, *bodkcm*, and *doapqr*) with *o* as the common vertex. Remarkably, Figure 1a also shows the charge density of the h-BN crystal by DFT calculations, and the black hexagonal VE encapsulates the full information on charge densities near the N atom at site *o* and its nearby three B atoms (at site *a*, *b*, and *d*); however, the green quadrilateral one fails to encapsulate the information. For perfect h-BN, B and N atoms are formed by covalent bonds with sp^2^ hybridization, while, for perturbed h-BN, they are formed by covalent bonds with deviated sp^2^ hybridization. Thus, a VE captures the correct ground-state bonding information near the center atom and a nonlinear response to the random perturbation can be conveniently obtained by the NN. In traditional tight binding theory,^48^ such perturbations can be captured by the so-called “bond orbital” coupling term, which is the major source of the correction term in our NN to the ground state. To improve the fitting accuracy for both energy and force, it is also possible to include more atoms in the VE as long as the ECM is satisfied. Also, VEs can be defined by considering each B atom as a center.

**Figure 1:**
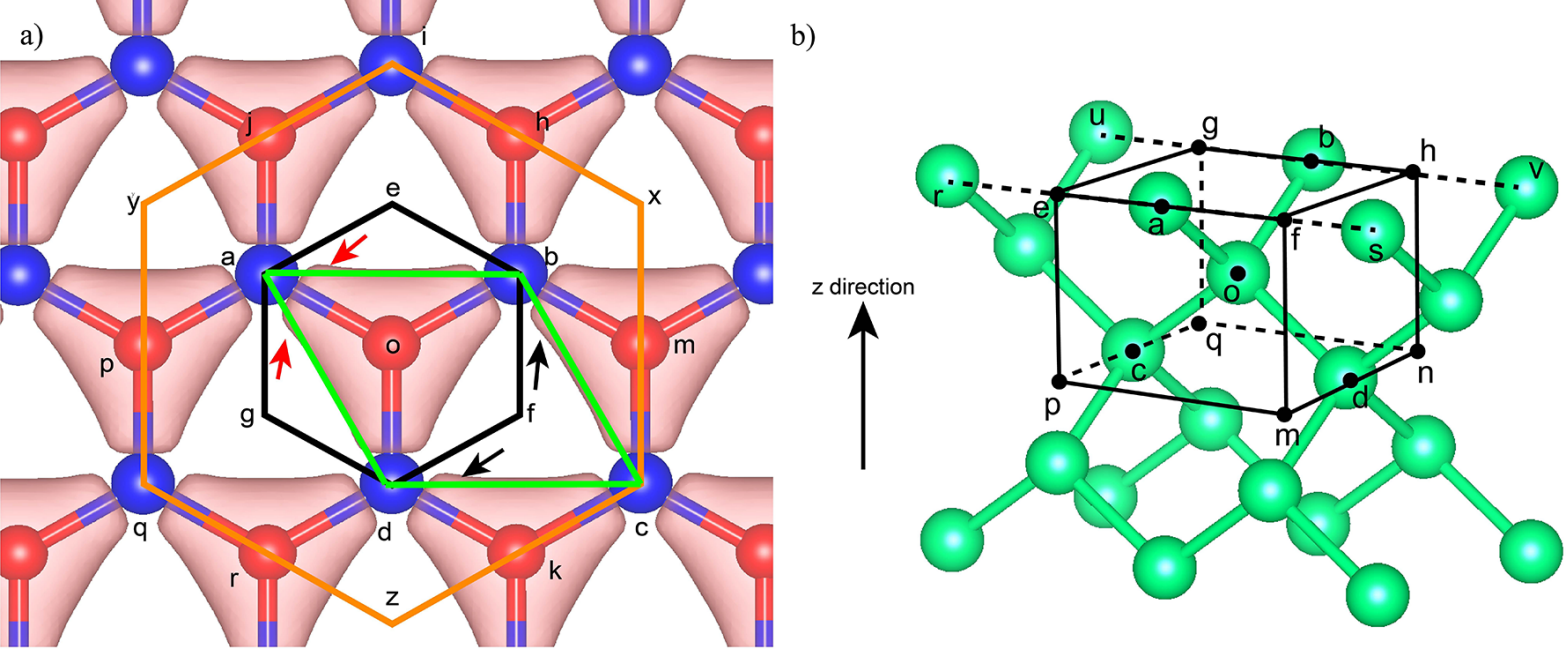
(a) Different types of volume elements of h-BN crystal, where blue spheres represent B atoms and red spheres represent N atoms. The green quadrilateral encloses *o* (N atom); *a*, *b*, *d* (three first-nearest-neighbor B atoms); and *c* (one-third-nearest-neighbor B). The black hexagon encloses *o* (N atom); *a*, *b*, *d* (three first-nearest-neighbor B atoms); and *e*, *f*, *g* (three geometric centers of nearby hexagons). The orange polygon is the expanded volume element. The charge density is shown as the pink shaded region. (b) Illustration of a volume element in bulk Si.

We can also construct VE for bulk Si, following a similar principle, as shown in Figure 1b. A Si atom, *o*, is placed in the center with four nearest neighbors, *a*, *b*, *c*, and *d*, placed at the centers of the four edges. The eight vertices (*e*, *f*, *g*, *h*, *m*, *n*, *p*, and *q*) at the corners are defined as the middle points between the edge center atoms and their nearby atoms with the same *z* coordinates. Such a VE is a primitive cell that is highly symmetric. Note that for a perturbed configuration the VE becomes a distorted polyhedron. For perfect bulk Si, each atom forms an sp^3^ hybridization with nearby Si atoms; however, when considering small random perturbations, the pure sp^3^ hybridization will couple with sp^2^ hybridization and form a mixture of sp^3^ and sp^2^ hybridization with neighboring atoms. Similarly, a VE captures the ground-state bonding information around the center atom, with the nonlinear response to the random perturbation easily obtained by our NN.

During training of our ML model, we transform the VE into a relative coordinate space with the center atom at the origin to satisfy the translational invariance. Additionally, a summation of energy from the VE as the output of ML satisfies the permutation invariance. At the inference stage, a rotational operation is applied to any VE inputs with the rotational axis at the center atom to align with an unperturbed VE. Once the maximum overlapping area is achieved, we use the corresponding coordinates as inputs of our pretrained NN to obtained the corresponding results, which satisfy the rotational invariance. See the Supporting Information for details.

A high quality reference data set is needed for training accurate ML potentials and force fields. To avoid the biased sampling approaches mostly based on optimized ionic structures,^49,50^ we adopted a random perturbation on a ground-state configuration and set the maximum perturbation intensity (MPI) as a key constraint. Additionally, the configurational population can be considered as a function of the total energy and should cover a certain energy range without any gaps. To obtain a continuous distribution, we can start from some trial sets and later add extra populations to fill up the gaps.

Our scheme is described as following: (1) Certain trial sets with different MPIs are generated from DFT calculations, e.g., 2%, 3%, 4%, and 5% of the unperturbed BN bond length, as shown in Figure 2. (2) Local distribution functions determined by MPI are approximated for the subsets, and in this case, we use Gaussian function as eq 1
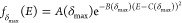
1where *E* is the total energy and the parameters *A*, *B*, and *C* are determined by MPI δ_max_. (3) Fit the corresponding parameters *A*(δ_max_), *B*(δ_max_), and *C*(δ_max_) and (4) estimate the overall population distribution by a weighted sum of the local functions with different MPIs as eq 2:
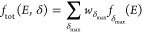
2where *w*_δ_max__ is the corresponding weight for each local distribution function. Note that the form of the local distribution function for any arbitrary systems can have arbitrary shapes, which may not necessarily be Gaussian-like. Guided by the estimated local distribution functions, a proper overall configurational distribution can be predicted theoretically by inserting more MPIs until continuity is reached (see the Supporting Information for details), and we summarize the whole data construction scheme in Figure 4.

**Figure 2:**
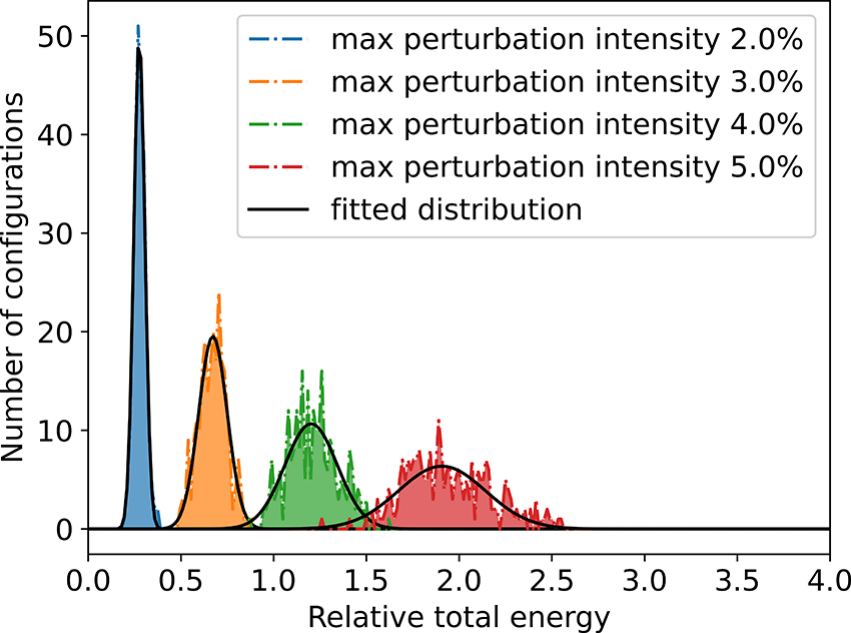
Four different maximum perturbation intensity distributions (dashed lines) in terms of energy and fitted Gaussian-like distribution (black lines) from DFT calculations.

**Figure 3:**
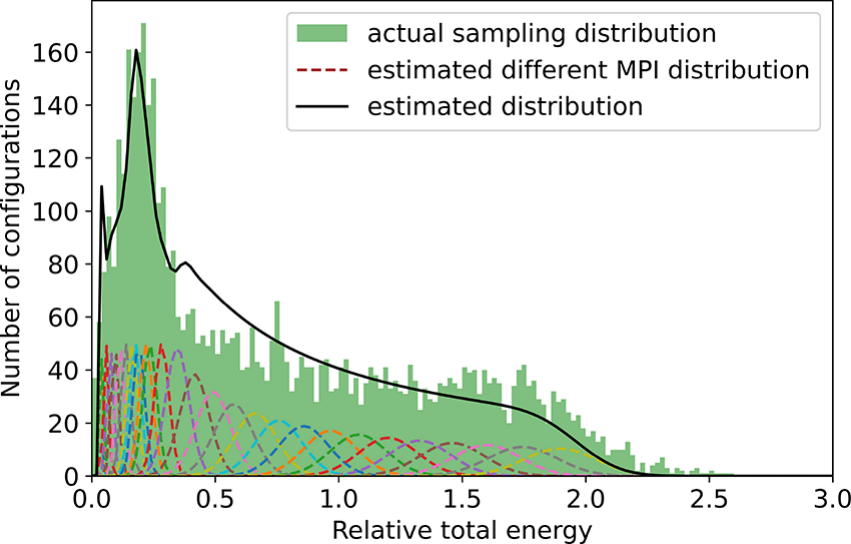
Estimated configurational distribution (black line) and constructed actual configurational distribution (green shaded region) in terms of energy (shifted by the ground-state energy); dashed lines label different maximum perturbation intensity (MPI) distributions.

**Figure 4:**
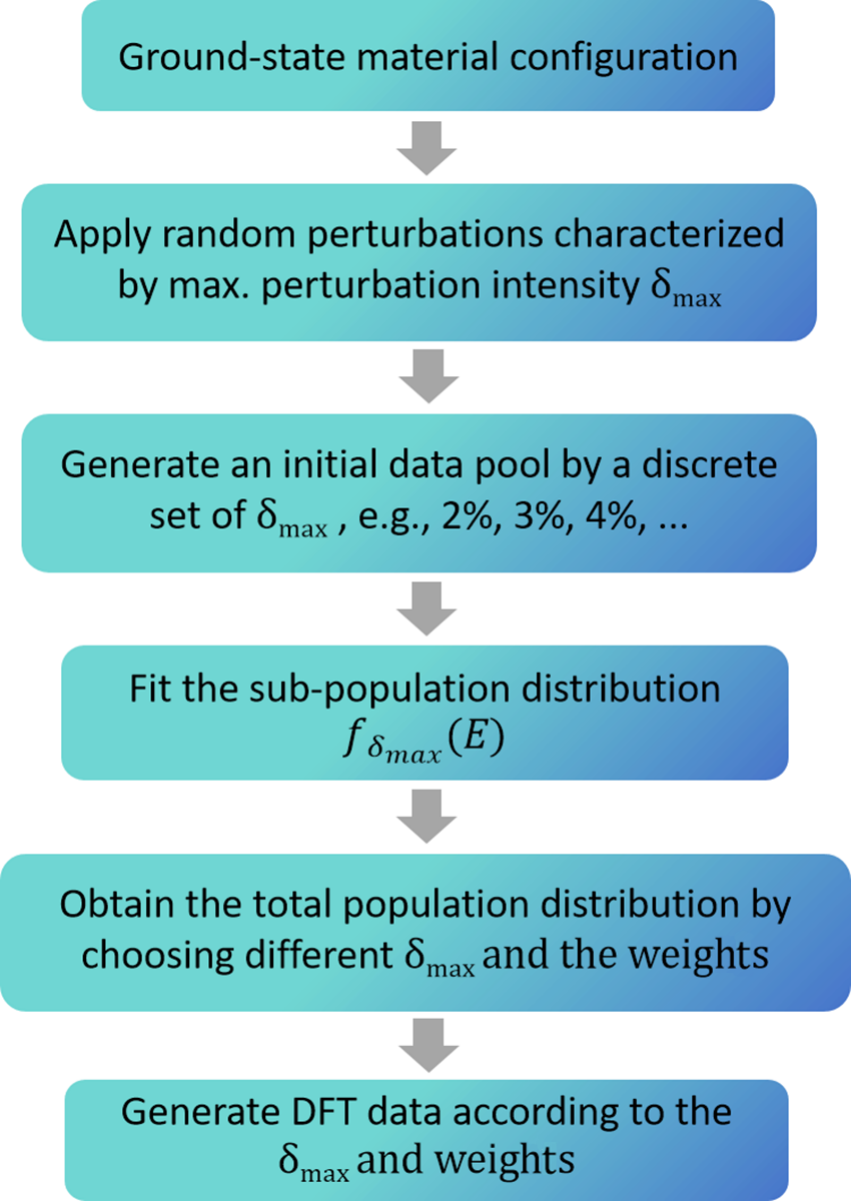
Summary of the data construction scheme.

As a result, Figure 3 shows an estimated continuous configurational distribution as a function of energy (black line) from eq 2 by adding different MPIs (dashed lines). Consequently, the actual sampling distribution shown in Figure 3 (green region) can be efficiently constructed by DFT calculations. Because of the limited computing resources, in the work, we only sample a continuous population distribution up to 5% MPI. However, in principle, it is possible to obtain a wider range of configurations efficiently by such an approach. In addition, we also conducted first principle MD at different temperatures, and the population distribution is sparse with high peaks (see the Supporting Information for details).

We adopted a simple fully connected NN that consists of one input layer, three hidden layers, and one output layer. Three neurons in the output layer output the “local energy” of each VE, as well as *x*- and *y*-component (for the 2D h-BN system) forces acting on its central atom, respectively. The total potential energy of a given configuration can be obtained by summing over all “local energy” contributions, i.e., . The activation function adopts a leaky rectified linear unit.^51^ As a regression problem, mean square error (MSE) loss is used to measure the distance between the predicted total energy and the DFT ones. The MSE of the predicted force for the center atom in each VE and the force of each atom in the DFT calculation are also calculated during force fitting. For the hyperparameter tuning, several efforts, such as ref (52), contribute to the acceleration of accurate selection of hyperparamters. The Adam^53^ optimization algorithm is adopted to minimize the total MSE loss with a dynamic adjustment of learning rate. See the Supporting Information for details.

The data set contains 4500 perturbed configurations of h-BN bulk materials from DFT calculations based on the aforementioned generation scheme, and the population distribution is shown in Figure 3. Each configuration contains 60 atoms. The configurations are processed into VEs and then passed into NN for training. 85% of the entire data set is split into a training set, and the remainder is a testing set. Note that we also tested a data set of 6000 structures that yield similar results. Also, our data size of the input set is about 1 order of magnitude smaller than any reported values in the literature^23,34^ and our approaches avoided difficult calculations of ionic movements in typical molecular dynamics and in principle should reduce computing resources additionally.

The accuracy of the VE method on the testing set is presented in terms of root-mean-square error (RMSE), and it has 0.51 meV/atom RMSE for potential energy. For the force RMSE, the VE method yielded 160 meV/Å. We showed that the force prediction accuracy can be further improved by expanding the VE with more atoms because force acting on each atom is very sensitive to its surrounding environment. We expand the VE by including three hexagons, as shown in Figure 1a (orange), and the expanded VE yields 0.57 meV/atom potential energy RMSE as shown in Figure 5 and 59 meV/Å force RMSE as shown in Figure 6. For the Si system, the simulation cell contains 72 atoms. We constructed 3800 perturbed configurations with an MPI of up to 3%. The VE approach yields a 0.43 meV/atom RMSE for potential energy and a 36 meV/Å force RMSE (see the Supporting Information for details), which demonstrate that our method is general for both 2D and 3D systems. We also compared the accuracy between our method and NequIP^54^ by retraining the NequIP (with *L* = 2) model on our data set under periodic boundary conditions, including both perturbed BN configurations and perturbed Si ones. Table 1 shows the accuracy of both energy and force RMSE. As a result, we observe that, for a perturbed BN system, our approach outperforms NequIP in energy predictions, and for a perturbed Si system, our approach outperforms NequIP in force predictions and with comparable energy prediction accuracy. This is probably because the Si system requires 3D volume element representations with one more degree of freedom that capture relative more surrounding information for each atom compared with 2D volume element representations of BN. The implementation details of NequIP can be found in the Supporting Information.

**Figure 5:**
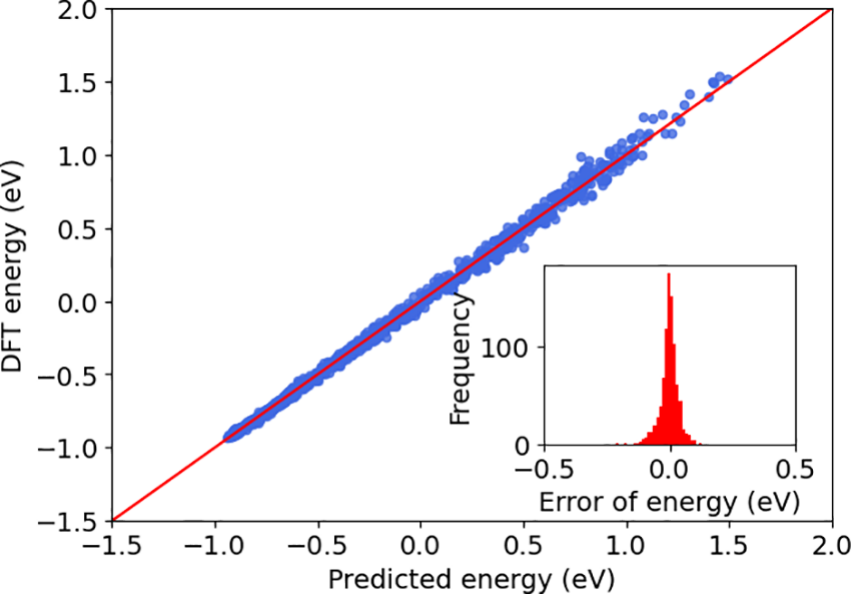
Training accuracy of potential energy with error distribution on testset. The energy is the relative energy to the ground-state configuration.

**Figure 6:**
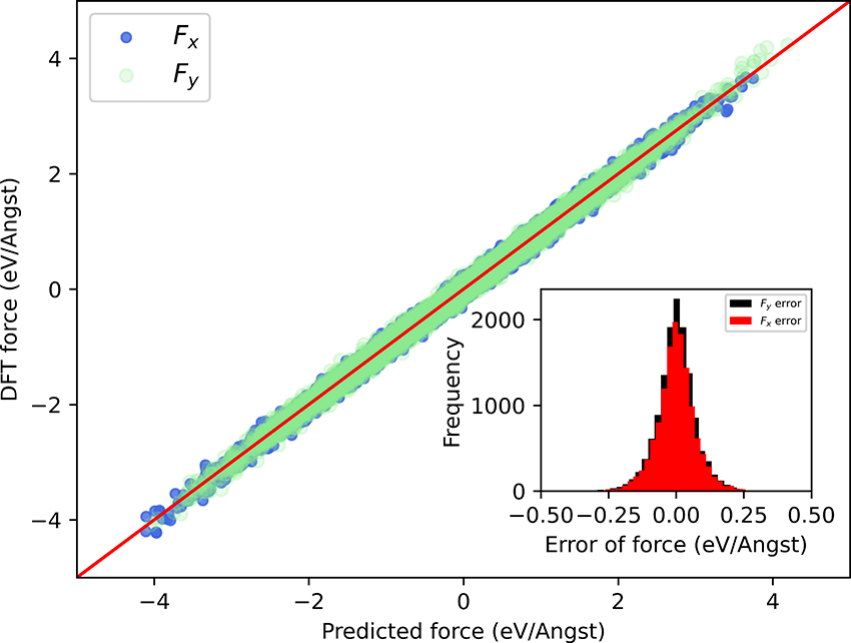
Training accuracy of forces with *x*- and *y*-component error distributions, where blue dots represent the *x*-component of force while green dots represent the *y*-component on the testset.

**Table 1:** Energy (*E*, meV/atom) and Force (*F*, meV/Å) Root-Mean-Square Errors on the Perturbed BN and Si Dataset for NequIP and Our Volume Element Method

Material System	Energy (*E*)/Force (*F*)	NequIP (*L* = 2)	Ours
Perturbed BN configurations	*E*	0.74	**0.57**
	*F*	**49**	59
Perturbed Si configurations	*E*	0.44	**0.43**
	*F*	47	**36**

We also compare the difference between our method and the traditional fixed radius methods.^34,35^ The fixed radius method defines a sampling unit by setting a random radius cutoff, which could easily miss important atoms on the boundaries and may result in an incomplete capture of charge density or incorrect estimation of total electrons that do not satisfy the ECM. However, for the VE method, we sample as much equal information as possible for each local contribution satisfying the ECM. Because adjacent VEs may share a common boundary or a few common atomic sites, the interaction between two VEs can be reflected by these coordinates. Therefore, the interaction energy terms can be absorbed into the self-energy term of each element, which is a function of the shape geometry and coordinates of the inner atoms. We also conduct a training based on sampling units with fixed cutoff radius (see the Supporting Information for details). As a result, the VE method yields the smallest RMSE for energy, and the expanded VE yields the smallest RMSE for forces. Although sampling units with increased radius cutoff contain more atomic information and thus may yield a better training result theoretically, the choices are still subjective and the ECM may not be satisfied because of the random perturbations. In addition, further optimization of the cutoffs is computationally expensive.

To verify the validity of trained potential energy and force fields in real applications, we adopt structure optimization with potential energy and force fields, given from our NN model. Specifically, gradient descent,^31,55^ steepest descent^56^ (SD), and particle swarm optimization^57^ (PSO) algorithms are all adopted to optimize structures with the 5% MPI into the most stable ones. During each iteration for GD and SD optimization, the new atomic positions are updated based on the predicted forces of the atoms in previous iterations from NN. For PSO, the particle velocities are initialized by initial forces, and in each iteration, it aims to lower the total potential energy predicted from NN. As a result, all of these algorithms successfully optimize unstable structures to stable ones under an error tolerance, i.e., 1.3 meV/atom for energy compared with DFT results (see the Supporting Information for details).

Another important application is MD simulation. We conduct the simulations with the potential energy and force fields obtained from NN from 100 to 400 K using a Berendsen thermostat in a canonical ensemble. The average total energy of VE-based and first-principles MD simulations is shown in Figure 7, where first-principles MD simulations are also conducted at the same temperature with the same thermostat. The average total energy from two approaches matches with the average 6 meV/atom of the average total energy difference below 400 K. Remarkably, the VE method is valid for the simulations from 100 to 400 K only based on a training of 4500 perturbed configurations that are significantly smaller than tens of thousand obtained from first-principles MD trajectories (see the Supporting Information for details). A heat capacity measurement at 300 K is also conducted by a multiple histogram method,^58,59^ and our simulation yields 35.6 J/(K·mol), which is comparable with experimental ones.^60^ Additionally, in order to verify the conservation of energy of our approach, a MD simulation in an NVE ensemble is also conducted for the BN system, and the results are in the Supporting Information.

**Figure 7:**
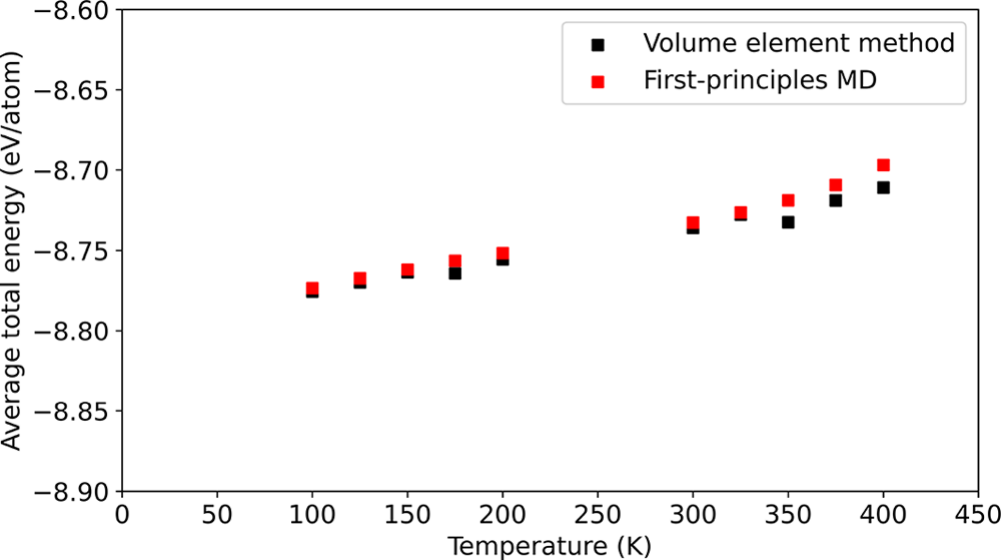
Average total energy per atom from molecular dynamic simulations by the volume element method (black squares) and first-principles calculations (red squares) in a canonical ensemble with a Berendsen thermostat.

Moreover, although the data set only covers a limited configurational population, we examine the extrapolation performance of our method. More specifically, potential energy and forces are predicted for some configurations with larger perturbations (5%–10% MPI). Our method can still give reasonable predictions for the configurations under 6.5% MPI with an average 1.3 meV/atom energy RMSE and 120 meV/Å force RMSE. Beyond 6.5%, the prediction accuracy gradually decreases (see the Supporting Information for details). Such extrapolation results also show good extensibility and stability of our method when dealing with outliers with relatively large perturbations. Additionally, the ground-state configuration can also be regarded as an extrapolation because it is not included in the training set and a 0.2 meV/atom energy RMSE and a 12 meV/Å force RMSE are obtained.

One of the valuable advantages for a typical accurate potential energy and force field ML model is the computation cost for large-scale systems compared with conventional first-principles or empirical force field MD. A direct force field prediction model processes almost linearly with an increasing number of atoms, which should be faster than gradient-based methods. We conduct a comparative study between our ML model and the first-principles MD for a relatively small simulation of 60 atoms and find that our ML approach is more than 2 orders of magnitude faster than the first-principles MD approach (see the Supporting Information for details). The VE approach can also be applied for simulations with large system sizes, and the enhancement should be more significant.

In summary, we proposed a parameter-free VE representation for accurate machine learning potential energy and force predictions. Such a representation differs from conventional bond length and angle models and enables satisfying ECM. In addition, a perturbation-based configuration construction scheme is proposed to capture a proper configuration population. Such a perturbation-based method includes stable, metastable, and unstable configurations without bias and enables rare configurations for typical long MD simulations to be captured, and thus improves the simulation stability and accuracy. Structure optimization, MD simulations, and extrapolations are also conducted to verify the validity of our method. Our VE approach also suggests a new representation to understand and describe quantum mechanical systems accurately and efficiently and may lead to future representations that are suitable for more complex systems.

## Supporting Information Available

Reference data set construction scheme; configurational populations comparison between the random perturbation scheme and the traditional scheme; permutational, translational, and rotational invariance; neural network architecture; improving force predictions by enlarging the volume element; accuracy for perturbed bulk Si; prediction accuracy comparison between the volume element method and the fixed random radius cutoff method; structure optimization; scalability and speed of volume element method; and extrapolations on configurations with stronger perturbations (PDF)
