# Supplementary material for: Parameter-Free and Electron Counting Satisfied Material Representation for Machine Learning Potential Energy and Force Fields
Source: J Phys Chem Lett. 2024 Feb 2;15(6):1636–43. doi: 10.1021/acs.jpclett.3c03250 (PMC10875669; doi:10.1021/acs.jpclett.3c03250)
Supplement: Supplementary file 1 — jz3c03250_si_001.pdf [file jz3c03250_si_001.pdf]

Supplemental Material: Parameter-free and Electron Counting Satisfied Material Representation for Machine Learning Potential Energy and Force Fields

Bin Xi, Man Kit Chan, Kejie Bao, Wenjing Zhao, Ho Ming Chan, Hang Chen, and Junyi Zhu*

Department of Physics, The Chinese University of Hong Kong, Shatin, New Territory, Hong Kong SAR

***Corresponding author:** jyzhu@phy.cuhk.edu.hk

I. **Reference dataset construction scheme**

We adopted a random perturbation on a ground state configuration of optimized hexagonal boron nitride (h-BN) and set the maximum value of the perturbation as a key constraint. We define the whole possible configurations based on the perturbation constraint as a phase space, and an ideal sampling set should cover most of the structures following the general distribution of the structures in the configurational space. Additionally, the structures can be considered as a function of the total energy and an effective population of structures as a function of their total energy should cover a certain range without any gaps. However, because the form of population as a function of random perturbations is unknown, it can be challenging to generate a sampling set with the same population of the potential configurational space.

One possible approach is to generate a trial set and investigate whether its population distribution follows the aforementioned requirement. One intuitive starting guess is to generate a continuous configurations distribution within a 5% maximum perturbation intensity (MPI). We first define a perturbation intensity δ for an atom as $\Delta r/l_0$, where Δr is the deviation of the atom from its unperturbed position and l_0 is the stress-free BN bond length. A first trial of the perturbation scheme is to generate configurations by setting different MPIs discretely, e.g., 2%, 3%, 4% and 5%. In Figure 3, four peaks of the population can be observed to represent the four different MPI distributions in terms of energy from DFT results (dash lines). There are gaps or very low population areas near the intersection region of four peaks. Note that for each peak distribution, it can be approximated by a Gaussian-like function (black

lines). Therefore, to mimic the overall distribution, we can first construct a sequence of Gaussian like local distribution functions determined by the MPI as equation,

$$f_{\delta_{max}}(E) = A(\delta_{max})e^{-B(\delta_{max})(E-C(\delta_{max}))^2}$$

Where E is total energy of each configuration and A, B and C are parameters to be fitted based on the four trails samples. The fitting results are shown in Figure S1. The overall population distribution can be defined as a function that sums up all the weighted local distribution functions as equation,

$$f_{tot}(E,\delta) = \sum_{\delta_{max}} w_{\delta_{max}} f_{\delta_{max}}(E)$$

where $w_{\delta_{max}}$ is the corresponding weight for each local distribution function. Note that, the form of the local distribution function for any arbitrary systems can have arbitrary shapes, which may not necessarily be Gaussian like. By choosing different MPIs, a continuous distribution of configurations can be approximated theoretically that is useful to efficiently generate a sampling space covering a large energy range continuously by DFT methods. In this work, 0.1%, 0.2%, 0.4%, 0.6%, 0.8%, 1%, 1.2%, 1.4%, 1.5%, 1.6%, 1.8%, 1.9%, 2%, 2.2%, 2.4%, 2.6%, 2.8%, 3%, 3.2%, 3.4%, 3.6%, 3.8%, 4%, 4.2%, 4.4%, 4.6%, 4.8%, 5% are used for MPI to sample a configurational population distribution as shown in Figure 4. To cover a wider energy range, the same scheme can be used by including more largely perturbed configurations (greater MPIs over than 5%).

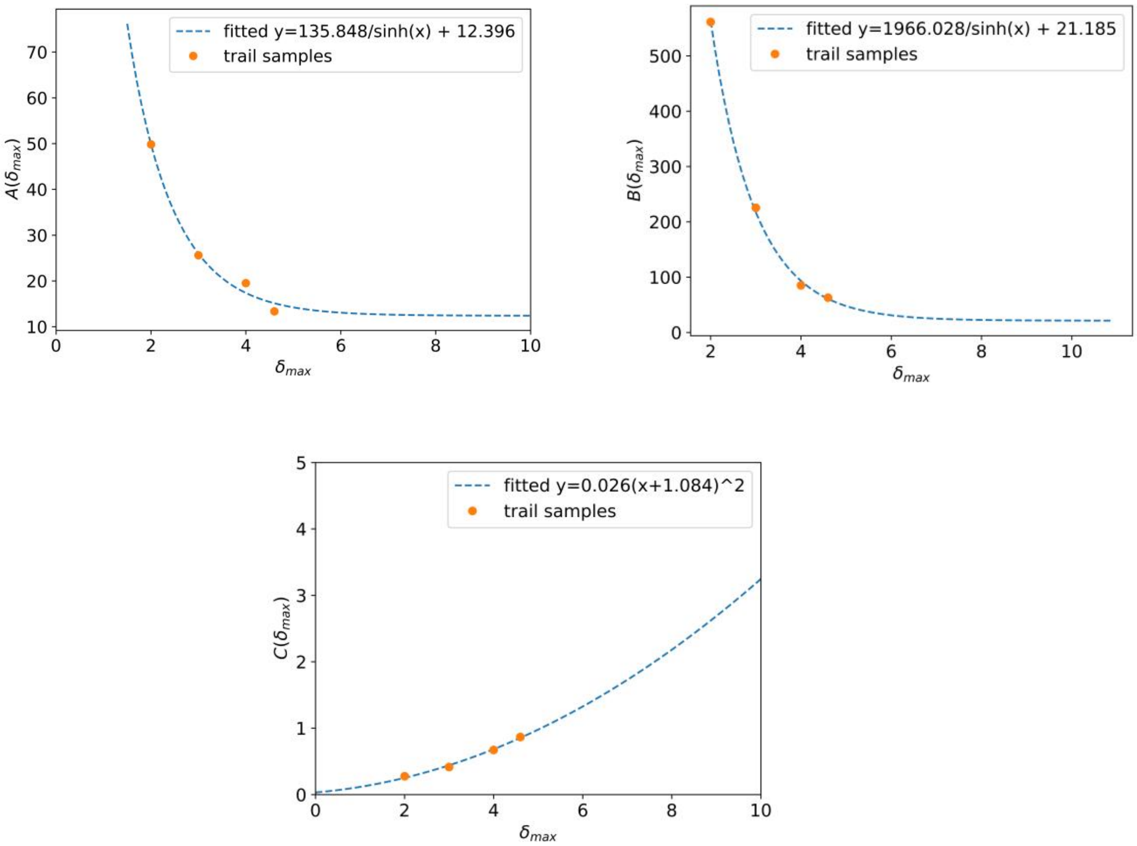

Figure S1: Fitted parameters A, B, C as a function of maximum perturbation intensity δ_{max} of Gaussian function, where orange dots are four trial sample points from DFT calculations and blue dash lines are fitted curves.

II. Configurational populations comparison between random perturbation scheme and traditional scheme

A reference dataset capturing as many configurations as possible is essential for training an accurate machine learning (ML) model. In the literature, many works adopted molecular dynamic (MD) simulations [1,2] or energetically local minima configurations from DFT calculations [3,4] to generate structures. Such approaches may suffer two issues: (i) the constructed dataset may not cover sufficient configurations and fail to reflect actual molecular motions; (ii) there may be some sharp peaks or gaps in the constructed dataset population. For this first issue, many intermediate configurations or unstable configurations can be missing, which could bring overfitting for the training of a ML model. Although, the idea of active learning [5,6] is adopted, the dataset may be highly sensitive to the initial sampling pool and remain biased and incomplete. Thus, a random perturbation scheme (see details in section I) is suitable for capturing stable, meta-stable and unstable configurations without bias.

For the second one, randomly combining different MD trajectories without any guidance may easily results in sharp peaks or gaps in the dataset population distributions. For example, we use first-principles MD to generate configurations at 250K, 300K and 350K in a NVT ensemble and we use the potential energy to characterize each configuration. Figure S2 shows three MD trajectories population distributions as a function of potential energy at 250K, 300K and 350K with a bin size 15meV. The energy is in reference to the ground state energy for illustration purpose. Each distribution is a Gaussian-like distribution and thus three sharp peaks are

observed, and gaps also exist in the range of 0eV to 1.3eV. The total number of configurations for the three populations are more than 40000, which however is still not able to capture a continuous total population distribution compared with our scheme shown in Figure 4. Note that, our dataset construction scheme only contains 4500 configurations which is much more computationally efficient.

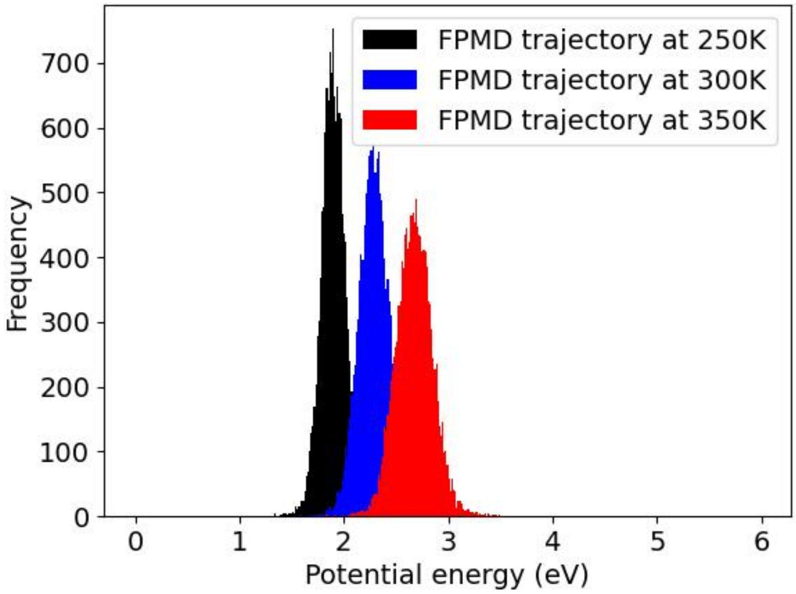

Figure S2: Population distributions as a function of potential energy for first-principles MD simulation trajectories at 250K, 300K and 350K with a bin size 15meV.

III. Permutational, translational and rotational invariance

A plausible ML model should be insensitive to permutational, translational and rotational operations. In the volume element (VE) representation approach, the summation over all "local energy" contributions from ML outputs, i.e., $E_{tot} = \sum_i E_i$ has preserved permutational invariance, while translational invariance is also naturally guaranteed because the inputs of ML model are relative VE vertices coordinates with the center atom as the origin. In addition, at the inference stage, we proposed a simple alignment approach to realize rotational invariance by taking the VE of h-BN as an example as shown in Figure S3. Specifically, a VE is pre-extracted from unperturbed ground state configuration as a template. And for a particular configuration with certain rotations, each of its VEs can be aligned with the template by coinciding the center atom coordinates of VE and that of template at the first stage. At the second stage, the intersection of union (IoU), which is defined as the factor of overlapping volume and the union volume, is computed and the IoU is maximized by applying rotations to the VE with the center as the rotation axis. Then, the aligned VEs can be inputs and evaluated by ML model.

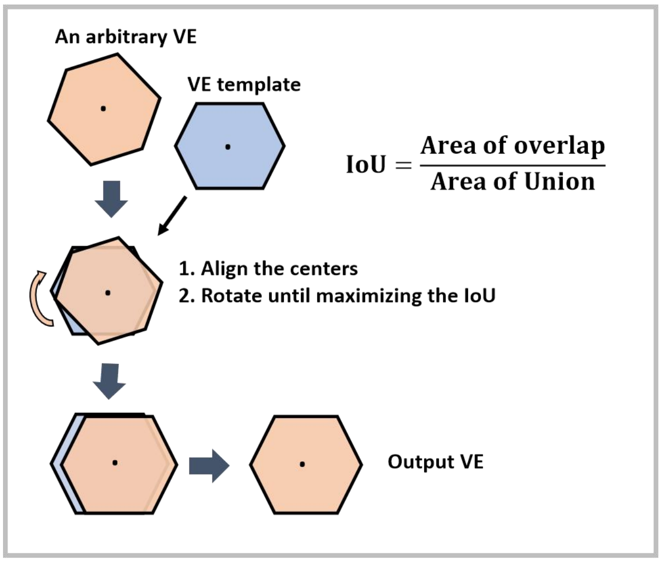

Figure S3: Schematic diagram of alignment approach to realize rotational invariance.

IV. **Neural network architecture**

In this section, we discuss the neural network (NN) architecture. A simple fully connected NN was adopted to process each VE, and the NN consists of one input layer, three hidden layers and one output layer. For each configuration, it is firstly processed into multiple distinct VEs. Then, the relative coordinates of the vertices on the VEs with their center atoms as origins are passed into the input layer. Hidden layers with different number of neurons collect the information from previous layer. Three neurons in the output layer output "local energy" of each VE, as well as x- and y-component forces acting on its central atom respectively, as shown in Figure S4. The total potential energy of a given configuration can be obtained by summing over all "local energy" contributions, i.e., $E_{tot} = \sum_i E_i$.

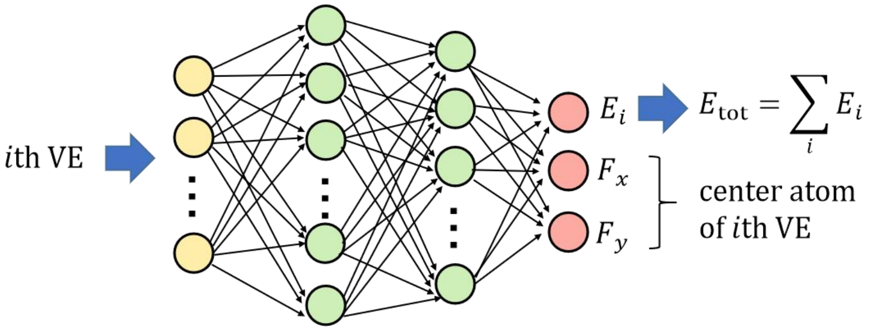

Figure S4: Schematic diagram of neural network architecture for processing one VE.

The activation function adopts leaky rectified linear unit [7] (leakyReLU) which are placed after neuron outputs to add nonlinearity for complex dataset. As a regression problem, mean squared error (MSE) loss is used to measure the distance between the predicted total energy and the DFT ones. The distance between the predicted force for

the center atom in each VE and the force of each atom in the DFT calculation is calculated as the inputs of the MSE loss for the force fitting. The loss for each configuration is defined as,

$$loss = p_e \left(E^{pred} - E^{DFT}\right)^2 + \sum_{i}^{N}\sum_{j}^{M}\left(F_{ij}^{pred} - F_{ij}^{DFT}\right)^2$$

Where p_e is a tunable prefactor, and E^{pred}, E^{DFT}, F_{ij}^{pred} and F_{ij}^{DFT} are predicted total energy, DFT calculated total energy, predicted force for jth component of ith atom and DFT calculated force for jth component of ith atom respectively. N is the total number of atoms for this configuration and M labels $\{x, y, z\}$ components. The Adam [8] optimization algorithm is adopted to minimize the total MSE loss with a dynamic adjustment of learning rate.

V. Improving force predictions by enlarging volume element

The VE representation as discussed in Methodology can be adopted to represent a given configuration objectively, by satisfying electron counting model (ECM) [9] without extra parameters. We take perturbed h-BN bulk materials as examples, and Figure S5 shows the a VE representation (black hexagon) for N centered atom. For the potential energy prediction from a ML model, such a VE representation includes sufficient information with one N atom (center atom at o site), one B atom (three vertices a, b and d) and the information of nearby VEs (three vertices e, f and g that are geometric centers of nearby VEs). The potential energy of a configuration can be obtained by summing over all the local "contribution" of VEs, and such a representation yields 0.51meV/atom root mean square error (RMSE).

However, for the force predictions, which is more sensitive to the surrounding atomic environments, we can expand the VE to include more atoms as shown in Figure S5 orange polygon. Such a bigger VE contains three N atoms, three B atoms satisfying ECM and also contains the information of nearby VEs (three non-atomic vertices x, y, z). Note that, the local geometry of the VEs is not only represented by the outmost atomic positions and average values of adjacent sites, but also by the positions of inner atoms. The expanded VE training results for both potential energy and force in terms of mean square error with error distribution are shown in Figure 5 and Figure 6. We also summarize the prediction results of the two VE representations from machine learning model in table SI.

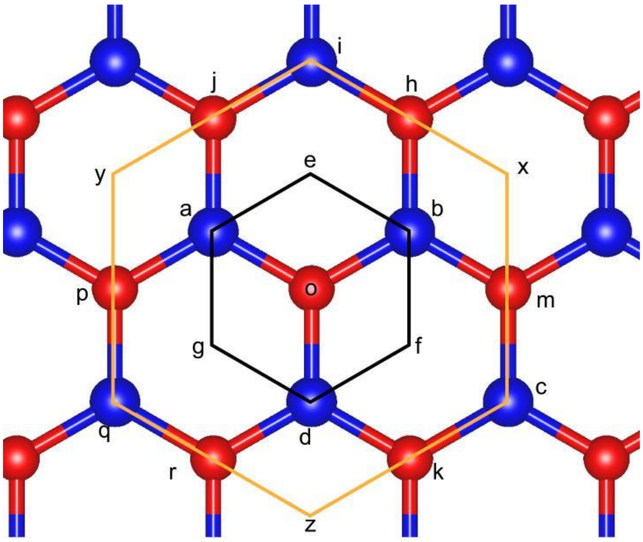

Figure S5: Small volume element representation (black) and expanded VE (orange) for h-BN bulk materials. Red spheres are N atoms and blue spheres are B atoms.

Table SI: Root mean square error (RMSE) for energy and force predictions. We take h-BN bulk materials as examples.

	Included atoms	Energy RMSE (meV/atom)	Force RMSE (meV/Å)
Volume element	1B and 1N	0.51	160
Expanded volume element	3B and 3N	0.57	59

VI. Accuracy for perturbed bulk Si

In order to verify the extensibility of the VE approach to three-dimension systems, we take perturbed bulk Si system as an example to show the success of our approach. Specifically, 3800 perturbed Si configurations are constructed from DFT calculations by following the data construction approach discussed in Methodology. Next, VEs are obtained as shown in figure 2. For the training of NN, we adopted three-hidden-layer architecture that is similar with the one for h-BN system, and a 85%-15% train-test split was used. As a result, it yields a 0.43meV/atom RMSE for potential energy and a 36meV/Å force RMSE.The detailed accuracy on the testset is shown in Figure S6.

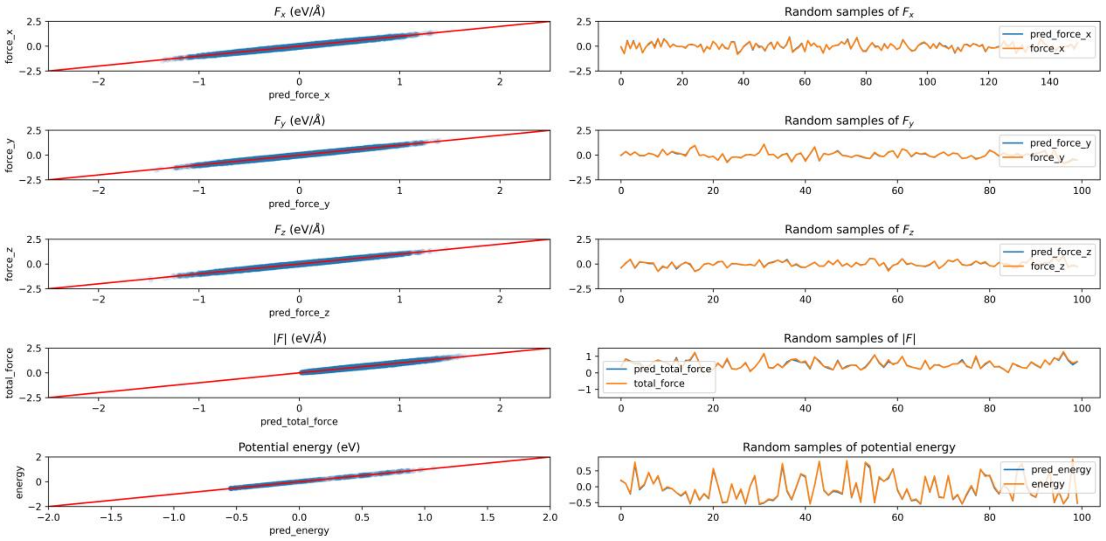

Figure S6: Potential energy RMSE and force RMSE of testset are shown in left column. Random samples are used to show the predicted potential energy and force with DFT results as shown in right column.

VII. Prediction accuracy comparison between VE method and fixed random radius cutoff method

In this section, we compare the difference between our method and the traditional fixed radius methods [2,10]. The fixed radius method defines a sampling unit by setting a random radius cutoff as shown in Figure S7, which could easily miss important atoms on the boundaries and may result in an incomplete capture of charge density or incorrect estimation of total electrons. However, for the VE method, we sample as much equal information as possible for each local contribution satisfying ECM. Because adjacent VEs may share a common boundary or a few common atomic sites, the interaction between two VEs can be reflected by these coordinates. Therefore, the interaction energy terms can be absorbed into the self-energy term of each element, which is a function of the shape geometry and coordinates of inner atoms.

We conduct a training based on a VE representation with fixed cut-off radius, as shown in Table S2. In this testing, VE representation method has the smallest RMSE for energy and the expanded VE representation has smallest RMSE for forces and 0.57meV/atom potential energy RMSE. However, the choice of cut-off radius will affect the training results with the worst case, i.e., 2 Å cutoff radius, having almost 3 times larger energy RMSE and over 3 times larger force RMSE compared with the best one. Although, VEs with increased radius cutoff contain more atomic information, thus may yield a better training result theoretically, the choices are still subjective and may capture redundant information and the ECM may not be satisfied because of the random perturbations, as shown in Figure S7. In addition, one may need to further

optimize the cutoffs based on training results, which are computationally expensive.

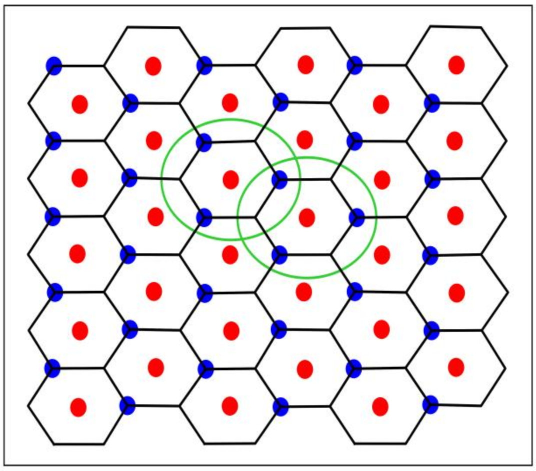

Figure S7: VE representation and random radius cutoff method of a perturbed h-BN crystal. Red dots represent N atoms while blue dots are B atoms. VEs are represented by black hexagons and any two adjacent area elements attach tightly with each other. A cutoff of 2 Å is chosen as green circles, which overlap with each other.

Methods	Energy RMSE (meV/atom)	Force RMSE (meV/Å)
VE	0.51	160
Expanded VE	0.57	59
2Å	2.11	354
2.75 Å	0.73	151
4 Å	0.90	89
5 Å	1.01	107

Table S2. Comparison of neural network training accuracy between VEs and random radius cutoff representations on h-BN bulk materials.

VIII. Implementation details of NequIP

We performed NequIP[11] experiments on our dataset, i.e., both perturbed BN and perturbed Si dataset, under periodic boundary conditions. We use the code from the original github and trained the NequIP model on single NVIDIA GeForce RTX 2080 Ti for almost two days with the officially suggested parameters in the documentation. Specifically, we use 4 layers with 32 channels for even and odd parity. We set the maximum irreducible representation order L=2, with a maximum 4 Å cutoff radius. 8 Bessel basis functions with polynomial envelope cutoff p=6 are used. The models were trained with a learning rate of 0.005 and a batch size of 5. The learning rate was reduced by using an on-plateau scheduler and AMSGrad optimizer was also used. The prediction accuracy can be found in Table 1.

IX. Structure optimization

We conduct test of structure optimization to verify the performance of our VE method. Specifically, for a given BN cell with random perturbations (5% MPI), gradient descent [12], steepest descent [13] are used to relax the cell into a stable one under 1.3meV/atom energy tolerance. Particle swarm optimization [14] is also used to obtain the optimal structure from 100 initial structures with random 5% MPI for each. During each iteration for GD optimization, the new atomic positions at time step $t+1$ are updated based on previous positions by following $\boldsymbol{R_{t+1}} = \boldsymbol{R_t} + \alpha \boldsymbol{F_t}$, where $\boldsymbol{F_t}$ is the predicted force from NN at time step t, and α is the step length that is 0.001Å^2/eV in this test. For SD optimization, the atomic positions for each atom are updated along initial force direction until the dot product of the current force predicted from NN and the initial one equals to zero. The energy difference of the optimized structure compared with the DFT relaxed result is 0.7meV/atom and 0.9meV/atom for GD and SD respectively. Figure S8 and S9 show the potential energy and mean force predicted from NN of the intermediate structures during the optimization for GD and SD algorithms respectively, where the dash line labels 0.02eV/Å.

For structure optimization by PSO, 100 structures with random 5% MPI are randomly selected as particles, and the initial velocity for each is set based on the force predicted from NN. During the optimization, the dth dimension of the velocity at time step $t+1$ for ith particle is updated by:

$$v_{id}^{t+1} = \omega v_{id}^{t} + c_1 r_1 (p_{id,pbest}^{t} - x_{id}^{t}) + c_2 r_2 (p_{d,gbest}^{t} - x_{id}^{t})$$

Where v_{id}^{t} is dth dimension of the velocity for ith particle at time step t, $p_{id,pbest}^{t}$ is the dth dimension of the personal best position for ith particle at t, $p_{d,gbest}^{t}$ is dth dimension of the global best position among all particles, x_{id}^{t} is the dth dimension of the ith particle position. While $\omega = 0.5$, $c_1 = 1.6$, $c_2 = 1.8$ in this test and r_1, r_2 are random numbers from 0 to 1. Later, the$d$th dimension of the ith particle position at $t + 1$ is updated by:

$$x_{id}^{t+1} = x_{id}^{t} + v_{id}^{t+1}$$

Figure S10 shows the potential energy of the global best position in each iteration. The energy difference of the optimized structure compared with the DFT relaxed result is 1.3meV/atom. Based on these optimization results, we show the success of our VE method for finding the stable configuration with given unstable configurations as inputs.

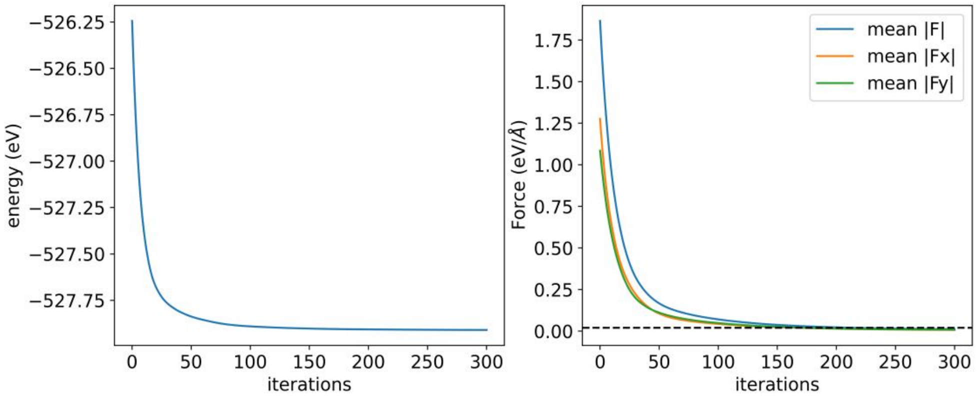

Figure S8: Structure optimization by gradient descent algorithm from neural network potential energy and force fields. (left) Potential energy and (right) mean force, mean x- and y- component of force for the intermediate structures during optimization, where the dash line labels 0.02meV/Å.

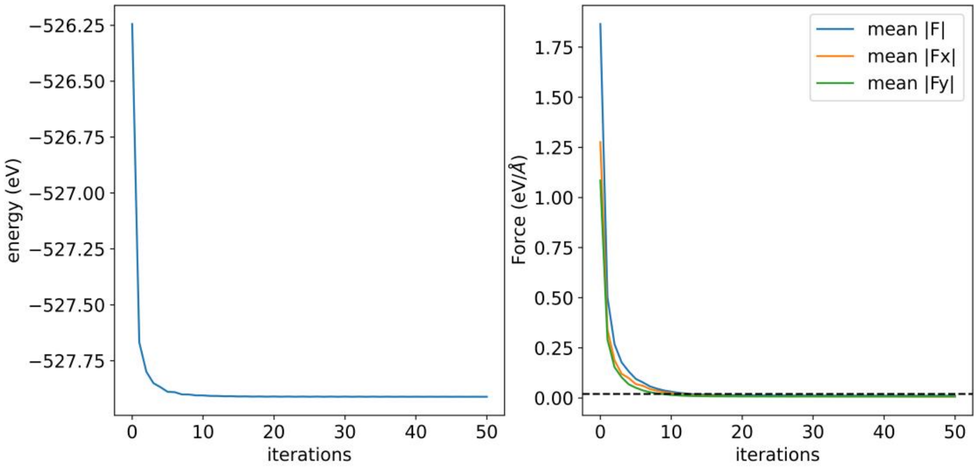

Figure S9: Structure optimization by steepest descent algorithm from neural network potential energy and force fields. (left) Potential energy and (right) mean force, mean x- and y- component of force for the intermediate structures during optimization, where the dash line labels 0.02meV/Å.

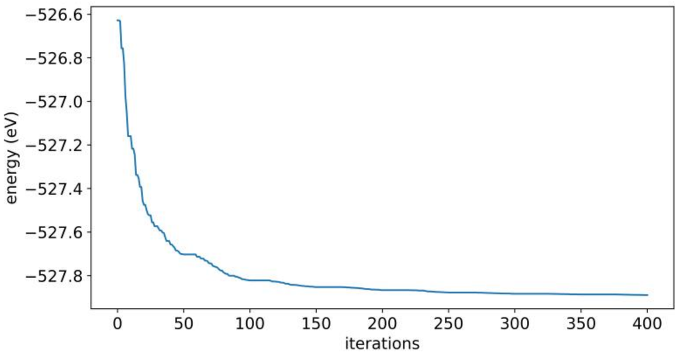

Figure S10: Potential energy during structure optimization by particle swarm optimization from neural network.

X. Scalability and speed of VE method

To verify the scalability of our method to large simulation cells, we conduct testing on 160- and 256-atom rectangular cells and 200-atom parallelogram cells under certain perturbations because our VE approach can be easily extended to these systems for both potential energy and force prediction. As a result, we obtain 0.47meV/atom energy RMSE and 65meV/Å RMSE for them, which show excellent scalability properties of our method.

The computation cost for such ML model scales linearly with system size, which is much faster than first-principles method. We conduct MD simulations of 60 atoms for 1.5ps to compare the computation cost of out ML method and first-principles MD in two nodes of 24 cores. As a result, the ML method is around 360 times faster than the first-principles MD. With the increase of system size, the difference can be more significant.

XI. Extrapolations on configurations with stronger perturbations

In this section, we discuss the extrapolation performance of our VE method on configurations with lager perturbations (larger than the largest MPI in dataset). The dataset is constructed by applying random perturbations on ground state configuration. Each configuration is characterized by its MPI (see methodology for details) and the entire dataset contains configurations with MPI $\leq$ 5%. To test the extrapolation performance of the ML model, we generate configurations with MPI lager than 5% and compare the prediction results of potential energy and forces from the ML model with DFT results. Specifically, configurations with 5.5%, 6.0%, 6.5%, 7.0%, 7.5% and 10% MPI are randomly generated. Figure S11 shows the prediction results of potential energy an forces in terms of RMSE versus different MPIs by VE representation (as shown by the black hexagon in Figure S5) and expanded VE representation (orange polygon in Figure S5). The dash line separates the coverage of training set configurations with MPI $\leq$5% and the extrapolations with MPI > 5% .

As a result, for potential energy prediction, the two kinds of representations yield similar results, while the expanded VE representation can improve the force prediction accuracy significantly because atomic forces are very sensitive to nearby atomic environment. In addition, the machine learning model can still give a reasonable energy prediction for extrapolations with the MPI in between 5% and 6.5%. The force RMSE shows an almost linearly ascend trend with the increasing of MPI even if the configurations are out of the training set space. In Addition, the

ground state configuration can also be regarded as an extrapolation because it is not included in the training set, and 0.2meV/atom energy RMSE and 12meV/Å force RMSE is obtained.

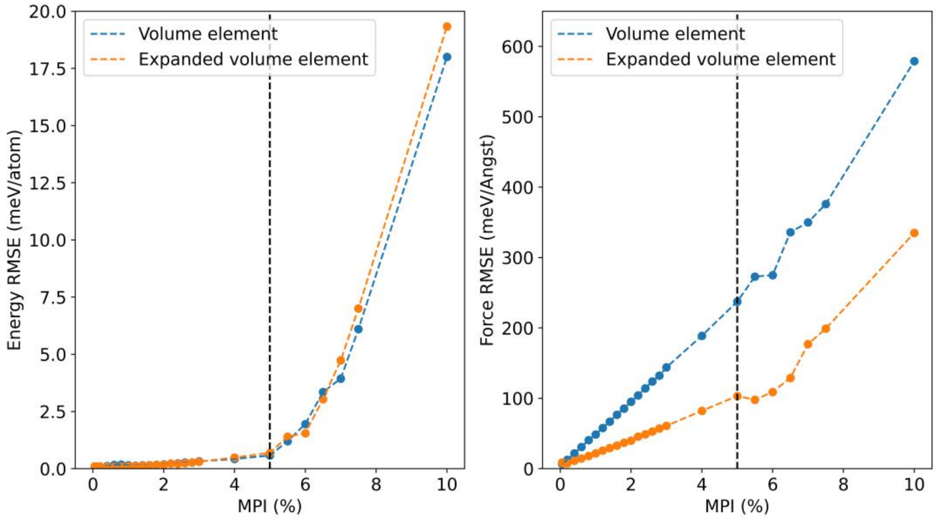

Figure S11: Potential energy and force RMSE from machine learning model by volume element representation (blue dots and dash lines) and expanded volume element representation (orange dots and dash lines). The black vertical dash line labels the marginal MPI (5%) of configurations covered in the training set.

XII. Verification of energy conservation by an NVE simulation

In order to verify the conservation of energy, we conduct a MD simulation in an NVE ensemble for a 60-atom BN system for more than 30 ps, which is equipped with the force fields from our volume-element machine learning model. Before the simulation of an NVE ensemble, a Berendsen thermostat is provided to heat the system to 400K by using an NVT ensemble. And after 10 ps, the system is assumed to reach equilibrium and the thermostat is then removed. Consequently, the simulation results of kinetic energy, potential energy as well as total energy as a function of simulation time are shown in Figure S12, where the red curves represent the NVT parts, while the black ones are the NVE parts, and the dash lines indicate the removal of the thermostat. The bottom plot of the figure shows the total energy is conserved nearly at -522.5eV during the NVE running, which indicates the conservation of energy by our approach. In addition, we compare the total energy result with the one of first-principle MD, the average total energy of the simulation of our approach is -8.703eV/atom, while the average total energy of first-principle MD is -8.698eV/atom, where they agree with each other with only 5meV/atom difference.

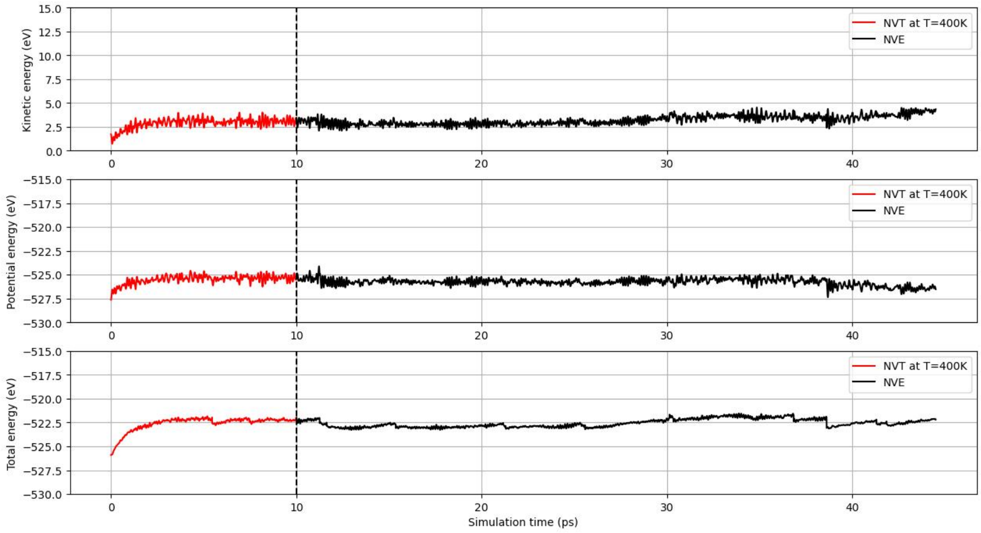

Figure S12. Plots of kinetic energy, potential energy and total energy as a function of simulation time in an MD simulation of 60-atom boron nitride system with an NVE ensemble. An Berendsen thermostat is added for heating the system to the equilibrium at 400K in an NVT ensemble (red curves) for 10ps. The thermostat is removed after 10ps (dash lines) and then the NVE simulation is running for more than 30ps.

Reference

(1) Chmiela, S.; Tkatchenko, A.; Sauceda, H. E.; Poltavsky, I.; Schutt, K. T.; Muller, K. R. Machine learning of accurate energy-conserving molecular force fields. *Sci Adv* **2017,** *3* (5), e1603015.

(2) Zhang, L.; Han, J.; Wang, H.; Car, R.; E, W. Deep Potential Molecular Dynamics: A Scalable Model with the Accuracy of Quantum Mechanics. *Phys. Rev. Lett.* **2018,** *120* (14), 143001.

(3) Hansen, K.; Biegler, F.; Ramakrishnan, R.; Pronobis, W.; von Lilienfeld, O. A.; Muller, K. R.; Tkatchenko, A. Machine Learning Predictions of Molecular Properties: Accurate Many-Body Potentials and Nonlocality in Chemical Space. *J Phys Chem Lett* **2015,** *6* (12), 2326-31.

(4) Rupp, M.; Tkatchenko, A.; Muller, K. R.; von Lilienfeld, O. A. Fast and accurate modeling of molecular atomization energies with machine learning. *Phys. Rev. Lett.* **2012,** *108* (5), 058301.

(5) Zhang, L.; Lin, D.-Y.; Wang, H.; Car, R.; E, W. Active learning of uniformly accurate interatomic potentials for materials simulation. *Phys. Rev. Mater.* **2019,** *3* (2).

(6) Smith, J. S.; Nebgen, B.; Lubbers, N.; Isayev, O.; Roitberg, A. E. Less is more: Sampling chemical space with active learning. *J. Chem. Phys.* **2018,** *148* (24), 241733.

(7) Maas, A. L.; Hannun, A. Y.; Ng, A. Y. In *Rectifier nonlinearities improve neural network acoustic models*, Proc. icml, Atlanta, GA: 2013; p 3.

(8) Kingma, D. P.; Ba, J. Adam: A method for stochastic optimization. *arXiv preprint arXiv:1412.6980* **2014**.

(9) Pashley, M. D. Electron counting model and its application to island structures on molecular-beam epitaxy grown GaAs(001) and ZnSe(001). *Phys Rev B Condens Matter* **1989,** *40* (15), 10481-10487.

(10) Behler, J.; Parrinello, M. Generalized neural-network representation of high-dimensional potential-energy surfaces. *Phys. Rev. Lett.* **2007,** *98* (14), 146401.

(11) Batzner, S.; Musaelian, A.; Sun, L.; Geiger, M.; Mailoa, J. P.; Kornbluth, M.; Molinari, N.; Smidt, T. E.; Kozinsky, B. E(3)-equivariant graph neural networks for data-efficient and accurate interatomic potentials. *Nat Commun* **2022,** *13* (1), 2453.

(12) Ruder, S. An overview of gradient descent optimization algorithms. *arXiv preprint arXiv:1609.04747* **2016**.

(13) Meza, J. C. Steepest descent. *Wiley Interdisciplinary Reviews: Computational Statistics* **2010,** *2* (6), 719-722.

(14) Wang, D.; Tan, D.; Liu, L. Particle swarm optimization algorithm: an overview. *Soft computing* **2018,** *22*, 387-408.
